# Advancing hypertensive disorders of pregnancy management: insights from the 5th preeclampsia scientific symposium in Ghana

**DOI:** 10.1186/s12919-024-00295-0

**Published:** 2024-06-13

**Authors:** Koiwah Koi-Larbi, Dorotheah Obiri, Joyce L. Browne, Linda Ahenkorah Fondjo, Samantha Katsande, Isabella Garti, On Behalf of APEC-Ghana

**Affiliations:** 1 Action On Preeclampsia Ghana (APEC-GH), Accra, Ghana; 2 Severe Pre-Eclampsia Adverse Outcome Triage (SPOT) Study, Accra, Ghana; 3 Julius Centre for Health Sciences and Primary Care, UMC Utrecht, Utrecht University, Utrecht, The Netherlands; 4 Department of Immunology, College of Health Sciences, Noguchi Memorial Institute for Medical Research, University of Ghana, Accra, Ghana; 5 Department of Molecular Medicine, School of Medical Sciences, Kwame Nkrumah University of Science and Technology, Kumasi, Ghana; 6 Faculty of Health, Charles Darwin University, Darwin, Australia

## Abstract

The 5th Preeclampsia Scientific Symposium (PSS2023) organized by Action on Preeclampsia (APEC) Ghana was themed*: ‘Realign, Refocus: Improving outcomes of Hypertensive Disorders of Pregnancy through Shared Decision Making, Research & Quality of Care’.* It took place on the 18th and 19th of May 2023 at the Ghana College of Physicians and Surgeons (GCPS), Accra Ghana. This transdisciplinary symposium brought together a national representation of experts, policy makers, scientists, and healthcare professionals to discuss key priorities, opportunities, approaches, and strategies to improve the maternal and perinatal outcomes of hypertensive disorders of pregnancy (HDP) in Ghana and the sub-region. The symposium centered around three key themes: realigning/refocusing patient-doctor decision making processes to improve outcomes of HDP; realigning/refocusing clinical care to improve outcomes of HDP; and leveraging on research to predict, recognize and manage high-risk women.

This report summarizes insights from the diverse presentations and discussions held at the #PSS2023. This will form a roadmap for future research, policy, and interventions to improve outcomes of HDP in Ghana and the sub-region. The symposium provided a wealth of evidence and knowledge from various experts, highlighting the need for women-centered care, equitable re-allocation of resources, multi-sectoral and innovative approaches, capacity strengthening. Other highlights include knowledge base development and increased stakeholder and community engagement with an overall aim of improving outcomes of HDP. The symposium also fostered inclusivity, welcoming survivors of HDP and their families at a scientific platform. They provided invaluable insights into the challenges faced and the lived experiences of those affected by the disease. Trainees and students also benefited from the symposium as it provided networking opportunities with fellow researchers, and a front row to gaining insights into cutting-edge research in Ghana.

## Highlights

• Nearing the completion of the SDG’s, engaging multi-sectoral and transdisciplinary perspectives, tools and systems is critical to improving treatment outcomes for women who develop preeclampsia in Ghana.

• Women play a pivotal role in decision-making regarding their care, and maternity services ought to cater to their unique requirements.

• Tackling the primary obstacles to delivering preeclampsia care in resource-limited settings holds significant promise for enhancing outcomes for both mothers and newborns.

• Research priority-setting offers a feasible approach to empower patients by improving their awareness, confidence, networks, and capabilities to engage in research, while also ensuring that their choices and preferences are valued and honored.

• The latest developments in predicting, preventing, and managing preeclampsia hold the potential to enhance outcomes for both mothers and babies in this life-threatening condition.

## Introduction

### Global burden of hypertensive disorders of pregnancy

Hypertensive Disorders of Pregnancy (HDP) remain a significant global health concern, contributing substantially to maternal mortality worldwide and impacting maternal, foetal, and neonatal health outcomes [1, 2]. These disorders include chronic hypertension, gestational hypertension, (superimposed) preeclampsia and eclampsia. The general features include elevated blood pressure during pregnancy with or without other end-organ damage [3, 4]. Globally, HDP are among the top 3 causes of morbidity and mortality amongst pregnant women [5] and affects about 5 to 10% of pregnancies [6]. Over the past 20 years, the incidence of HDP has increased by more than 10% globally from 16.30 million and now affecting 18.08 million women [2]. Preeclampsia is the most prevalent variant linked with adverse foetal effects such as intrauterine growth restriction, premature birth, placental abruption, foetal distress, and stillbirth [7]. Survivors of preeclampsia, especially the severe and early onset forms, are at higher risks of cardiovascular diseases later in life [8, 9].

### HDP in Ghana

In Ghana, approximately 25% of maternal deaths are attributed to HDP, particularly in tertiary hospitals across the country [10]. Among the HDP, preeclampsia is the leading contributor to maternal mortality across major tertiary hospitals in Ghana [11–13]. Given the heightened level of morbidity and mortality, there is a pressing need for interventions aimed at enhancing the quality of care for women diagnosed with hypertensive disorders of pregnancy (HDP).

### Patient advocacy and support: action on preeclampsia- Ghana

Action on Preeclampsia (APEC) Ghana is a national HDP patients’ and survivors’ advocacy organization in Ghana, and consists of a multidisciplinary network of survivors, researchers, (including medical and social scientists), and healthcare practitioners. Since its inception in 2017, APEC has provided a platform to address issues related to HDP in Ghana. APEC aims through its annual scientific symposium organized to commemorate World Preeclampsia day, to promote dialogue among stakeholders from across various sectors, to advance science-based solutions, spur research interventions and encourage multi-sectoral collaborations to improve HDP-related healthcare and health outcomes. To integrate local and global knowledge, APEC’s goal is to shape a transdisciplinary national HDP research agenda, provide evidence-informed recommendations to optimize healthcare practices, policies, and programs for the effective management of HDP in Ghana. Through its research and scientific committee, APEC continually works to generate and disseminate insights and interventions that are shaped by data-driven research and rooted in on-the-ground, community perspectives towards action-oriented solutions to improve outcomes of HDP.

### 5th Preeclampsia Scientific Symposium (*PSS2023*)

In May 2023, APEC convened its 5th Preeclampsia Scientific Symposium (*PSS2023),* themed *Realign, Refocus: Improving outcomes of Hypertensive Disorders of Pregnancy through Shared Decision Making, Research & Quality of Care.* This transdisciplinary hybrid meeting brought together a national representation of experts including healthcare professionals (midwives, nurses, resident doctors and consultants in obstetrics/gynecology), policy makers, and researchers from the biomedical and social sciences, trainees and students to discuss key priorities, opportunities, approaches and strategies to improve the outcomes of HDP in Ghana and the sub-region. The event saw participation and in person attendance of more 250 participants and a virtual attendance exceeding 80 participants. The symposium participants came from diverse geographical locations, spanning across all 16 regions of Ghana.

International representation included participants from the Netherlands, Nigeria, the United States, the United Kingdom, and Australia. There was a formal call for abstracts, and all submitted abstracts went through a blind review process.

During the two-day symposium, there were four plenary presentations, nine parallel sessions, three oral presentations, six poster presentations, four keynote presentations from partners, and a concluding roundtable panel discussion centered around the conference themes.

The PSS2023 symposium centered around three key themes reflected in the conference title, and stemming from the following questions:

I. *“How can patient-doctor decision making processes be realigned/refocused to improve outcomes of HDP?”*
II. “*How can clinical care be realigned/refocused to improve outcomes of HDP?*”
III. *“How can maternal health research be realigned/refocused to improve outcomes of HDP?”*

In this report we provide a thematic overview of the primary insights from the symposium presentations and discussions regarding strategies for enhancing the quality of care through research and shared decision-making to improve outcomes of HDP. Each theme is thoroughly examined in its dedicated section.

## Emerging themes

Below we present key insights per theme:

### Theme 1: realigning/refocusing patient-doctor decision making processes to improve outcomes of HDP

Understanding the concepts of respectful maternal care, risk communication, shared-decision making, and the quality improvement cycle is vital for the implementation of impactful solutions. The key learnings related to this question were that first, Respectful Maternity Care (RMC) is a human right issue vital for the achievement of good maternal health outcomes. Second, a global approach that supports effective and sustained implementation of evidence-based care for routine, antenatal, intrapartum and postnatal care is urgently needed [14]. And third, ‘full’ shared decision making, a key element of RMC may not be feasible within the context of HDP care provision in Ghana, however, incorporating some components is important and requires addressing patients' concerns and building trust. The role of patients in the quality improvement cycle cannot be overlooked.

#### Respectful maternity care

According to the World Health Organization “*every woman has the right to the highest attainable standard of health, including the right to dignified, respectful care during pregnancy and childbirth*.” [15]. Respectful maternity care (RMC) is essential for women with or at risk of preeclampsia, as it fosters shared decision-making, may encourage acceptance of diagnosis and treatment, and cultivates a collaborative relationship between patients and healthcare providers, thus, addressing their concerns more effectively. Dr. Kwaku Asah-Opoku emphasized this need in the parallel session on Respectful Maternal Care: working together for impact to improve outcomes of HDP. The components of RMC were outlined to include being free from harm and mistreatment, maintaining privacy and confidentiality, preserving women’s dignity, prospective provision of information and seeking informed consent [16]. Other components include ensuring continuous access to community support, enhancing quality of physical and environment resources, engaging with effective communication, and respecting women’s choices that strengthens their capabilities to give birth. The availability of competent and motivated human resources is also very necessary for the provision of RMC.

Implementing respectful maternity care (RMC) throughout the patient's journey, including diagnosis, treatment, birthing, discharge, and post-discharge follow-up, is crucial for improving outcomes in hypertensive disorders of pregnancy (HDP) [17]. For example during diagnosis, it is essential to consider factors like whether the diagnosis and its implications were adequately explained to the patient in a manner she could understand, and whether there was the presence of a companion or professional support [18]. The challenges of and barriers to RMC negatively impact patient experiences and provision of quality care. These barriers include entrenched position of caregivers, language barriers (homogenous languages in most HICS, heterogenous languages in LMICs), such as difficult translation of terminology including disease name (preeclampsia has no local name in Ghana), limited facilities for alternate options especially in LMIC’s and, Health service provision inequities [19, 20].

Experts at the conference highly recommended that to promote RMC at both facility and policy level, simulations, and team-training on emergency obstetric and neonatal care as part of management of HDP should consciously have respectful maternity care component. Moreover, the training environment should mirror the intricate and demanding conditions characteristic of regular work settings. There should also be continuous and increased institutional and public education on RMC, corresponding protocols that include shared decision making and RMC components, systematic RMC audits, and operational research on how providers can provide RMC to improve patient experiences and maternal and perinatal health outcomes. RMC is important for the achievement of optimal outcomes and birth experiences, and as such strategies should be explored to ensure that women with HDP receive RMC.

#### Risk communication & shared decision making

Respectful Maternity Care is a facilitator of shared decision making. A key element of this process is Risk-communication—the process of communicating risk of the disease and associated treatment [21]. “*How much information is too much ? How much information is too little ?.*” Dr. Bregje de Kok of the SPOT-Impact Study in her session “Risk Communication & Shared Decision Making” elucidated the function of risk- communication by sharing the findings of the Exploration Phase of the Co-Creation of the SPOT Toolkit. A tool-kit to support the provision of care for women with HDP is currently being developed co-creatively by academic and non-academic stakeholders including health care professionals, policy-makers, patients and survivor and is expected to be implemented and evaluated in 2024.

**Fig. 1:**
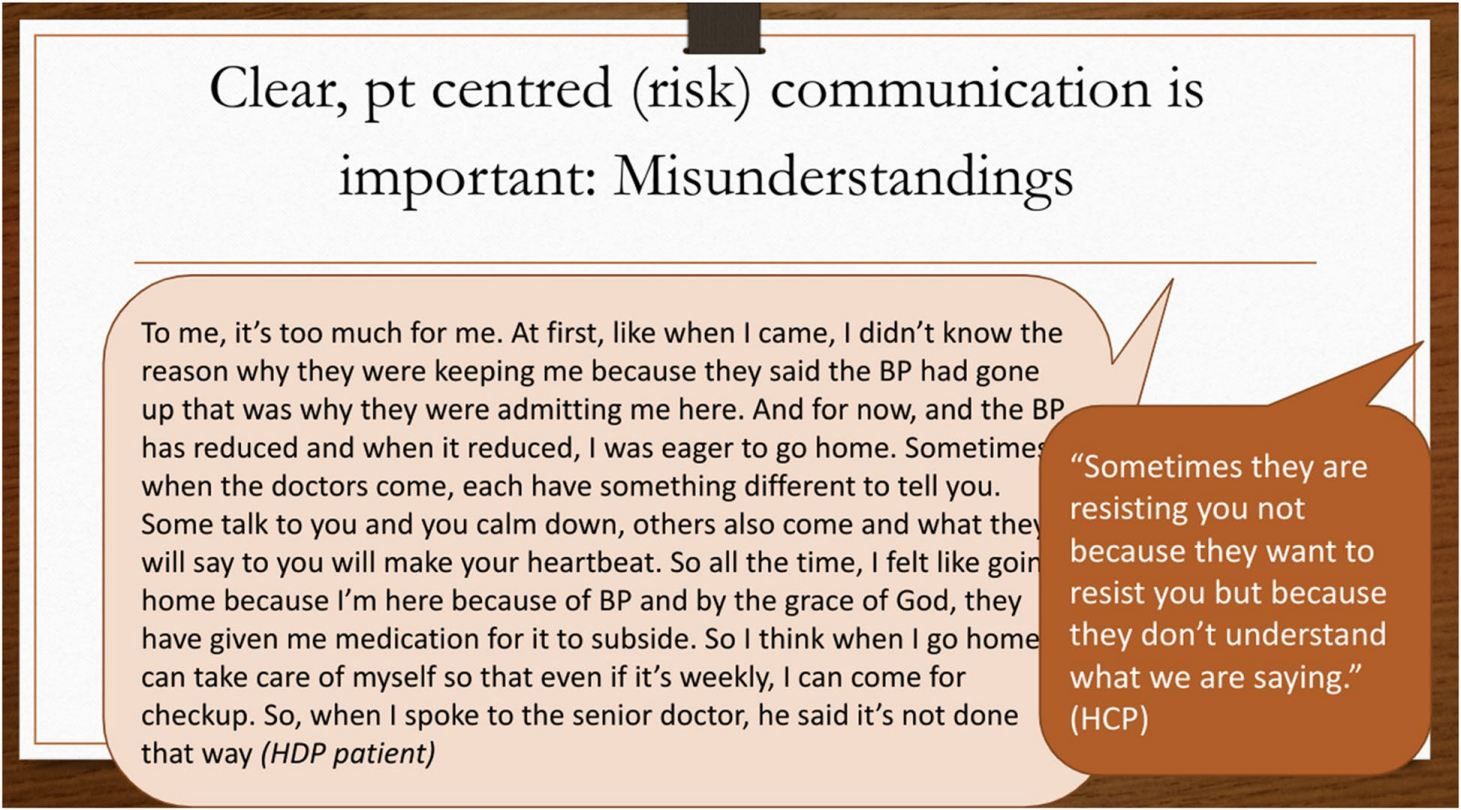
Patient and health care provider experiences with risk communication. Pt: patient. HCP: Health Care Provider

As a consortium, the SPOT Study adds onto the definition of shared decision making to include ‘*the extent to which a patient is involved in the final decisions albeit dependent on the maternal and fetal conditions and context. Regardless, information provision and informed consent is essential and always need to be ensured*’ and identifies risk-communication as an important precursor. Although the findings acknowledged that this is not always possible including during emergencies or when patients are unwilling to engage, it was recommended that patient-centered communication and decision-making should be utilized. Thus facilitators of shared decision making were identified to include communication skills, use of graphics, building a good relationship and rapport, offering reassurance and a message of hope. The barriers to shared decision making were categorized as follows: the disease factors (the urgency); provider factors (personality, attitude, fears about accountability /litigation); health system and organizational barriers ( workload, lack of privacy, language barriers, lack of continuity of care- conflicting messages, facility management leadership); client factors (education level, health literacy); and provider–client relationship factors (sub-optimal trust and information flow). Evidently, the importance of patient-centered risk communication and the impact of misunderstanding on patient reaction was also revealed. Figure 1 captures patient and health care provider experiences with risk communication from the findings shared by Dr. de Kok.

The findings of the exploratory phase of the SPOT toolkit showed that in Ghana shared decision making is valued primarily as a means to encourage patients to accept clinicians' recommendations. Hence achieving mutual understanding, addressing patient concerns, and building trust is important. Full shared decision making may not be feasible when dealing with HDP clients but there is still room for patient-centered communication that empowers patients, addresses their needs, and is based on the patient’s values, wishes, personal circumstances [22].

#### Quality improvement cycle: the role of patients

The SPOT study findings undeniably signify that patients play a central role in quality improvement and hence should be empowered, supported, and involved in the various stages of the quality improvement cycle to improve outcomes of pregnancies [23]. They should be involved in audit (implementation), included in randomized controlled trials on medication & interventions, involved in research and education, involved in guideline development and in development of research agenda. These key points were emphasized by Prof. Bloemenkaemp in her presentation “Quality Improvement cycle- The role of patients to improve outcomes of HDP.” To emphasize this call, it was highlighted that the 2025 EPMM Goal is to have about 65% of women aged 15–49 make their own informed and empowered decision regarding sexual relations, contraceptive use and reproductive healthcare [24]. Increasing education, improving health literacy, and generating awareness among pregnant women about alarm symptoms in pregnancy, especially regarding hypertensive disorders and cardiac symptoms, is an effective way to support women in these roles.

### Theme 2: realigning/refocusing clinical care to improve outcomes of HDP

A recurrent theme at #PSS2023 was the reality that “ there are pervading challenges with clinical practice in resource- constrained settings in LMICs such as Ghana. The analysis involved examining the quality of various clinical practices in antenatal and postnatal services using case studies, evaluating the delivery of emergency obstetric care in Ghana, assessing innovative tools for improving sick and newborn care, examining drug-use principles in the management of hypertensive disorders of pregnancy (HDP), and evaluating the availability of HDP commodities in the Public Health Sector. Some of the key learnings related to this theme were that several barriers exist throughout the continuum of pregnancy care that limits the early identification and care for Ghanaian women with HDP; in Ghana, a strategy-oriented approach to measuring readiness is needed to reduce facility-based obstetric mortality; the obstetric pharmacist is an integral cadre in hypertension management and that accurate medicine reconciliation is crucial at transitions of care between wards or in the clinic. On a macro-economic level, high inflation drives the cost of HDP commodities beyond levels that can be sustained under the National Health Insurance Scheme (NHIS). Also, inadequate stock levels affect clinical care for women with hypertensive disorders of pregnancy.

#### Quality of maternity care services- delivery of emergency obstetric care in Ghana

Emergency obstetric and newborn care (EmONC) facilities address major obstetric complications like preeclampsia through medical treatments and manual procedures [25]. In a presentation by Dr. Henry Kintoh Kumi, it was revealed that despite the significant occurrence of preeclampsia and its associated fatalities, the efficient utilization of the EmONC system remains elusive, leading to delays in recognizing and treating preeclampsia. The World Health Organization (WHO), and other stakeholders in maternal and child health advocate for emergency obstetric care (EmONC) to manage major obstetric complications effectively [26]. In this model facilities are categorized as Basic or Comprehensive EmONC based on their provision of specific life-saving services known as signal functions. These functions include both medical treatments (administration of antibiotics, anticonvulsants, and oxytocics) and manual procedures (assisted vaginal delivery, removal of retained products of conception, and retained placenta) [27]. The readiness of facilities to manage obstetric emergencies is assessed using the service readiness index, which measures the presence of 11 tracers. However, current readiness estimates based solely on signal functions fail to account for the sequential and simultaneous resource requirements for clinical management.

Standard signal function estimates and the WHO’s obstetric service readiness index do not measure readiness for each clinical disorder. If we estimate emergency-specific readiness using the signal function tracer items alone, many resources required to practically deliver care would not be accounted for. For example, signal function estimates for eclampsia would be defined as the proportion of facilities with IV solution/infusion sets, hydralazine, and magnesium sulphate.

The clinical cascade model offers a more nuanced approach to measuring emergency readiness by emphasizing the interconnectedness between resources needed to diagnose emergencies, administer treatments, and monitor patients' responses [28]. This model reveals discrepancies between the availability of maternal signal function tracer drugs and the resources necessary to identify and treat emergencies effectively. To enhance emergency obstetric and newborn care, EmONC facilities in Ghana and other LMICs must support skilled attendants working in communities, recognizing the critical link between skilled care and an enabling environment. Improving EmONC requires accreditation, adequate funding, continuous training, and effective leadership. Stakeholders must assess the readiness of health facilities to handle preeclampsia by ensuring the availability of basic supplies, skilled personnel, and essential medications. A strategy-oriented approach is necessary to drive reductions in facility-based preeclampsia mortality in Ghana. Stakeholders need to regularly assess and ask about health facilities' basic supplies, skilled personnel, availability of antihypertensives and anticonvulsants, and patient-centered delivery of HDP services to ensure comprehensive readiness for managing hypertensive disorders of pregnancy effectively.

#### Drug-use-principles in the management of HDP: the role of the obstetric pharmacist

Teratogenicity in obstetric pharmacology is a concern due to potential anatomical or functional issues in the developing foetus, especially in preeclampsia and hypertensive disorders [29]. Factors like dosage, timing, administration routes, genetics, and environmental influences impact foetal development positively. Hypertensive disorders of pregnancy call for polypharmacy, which raises concerns about potential teratogenic consequences. In managing preeclampsia, preventing seizures, and controlling hypertension are paramount, with magnesium sulphate recommended for seizure prevention [3, 30]. Antihypertensive choice is often influenced by efficacy and safety in alignment with recommendations in context specific clinical guidelines [29]. Antihypertensive choice depends on efficacy, safety, and gestational age, with prompt administration preventing complications. Magnesium sulphate, effective in seizure prevention, requires cautious administration due to potential complications. Certain medications like diuretics and Angiotensin converting enzyme inhibitors are not recommended for use in pregnancy and should be avoided due to potential foetal harm [3, 31].

Close observations are necessary to ensure optimal management and minimize potential risks to both the mother and the foetus [3]. Women with pre-existing hypertension should transition from current medications like angiotensin converting enzyme inhibitors and angiotensin II receptor blockers to alternative options. Post-delivery, treatment may persist, with regular blood pressure monitoring, ideally utilizing home monitoring [3, 30].

Dr. Marc Dzradosi elaborated that the obstetric pharmacist is well equipped to enhance maternal and foetal wellbeing [32]. This role involves evaluating the risks and benefits for each individual patient thereby ensuring physicians are mindful of the dosage, timing, and co-administration of drugs [32, 33]. In addition, clinical pharmacists in a multidisciplinary establishment may contribute to hypertension management through patient education and counselling, and in-hospital and post -discharge follow-up monitoring. However this role may be challenged by knowledge gaps, supply chain issues, and a lack of adherence to protocols, highlighting the importance in addressing these obstacles especially in LMICs [34, 35]. Moving forward, it is essential to strengthen interdisciplinary collaboration and address healthcare system challenges as these are crucial for improving obstetric care globally, particularly in LMICs, where access to specialized care and resources may be limited. By implementing comprehensive guidelines, integrating pharmacists into multidisciplinary teams, and addressing barriers to care, LMICs can enhance maternal and foetal health outcomes, ensuring optimal care regardless of resource constraints.

#### Sick and newborn care

Dr. Emmanuel Parbie Abbeyquaye's presentation on ‘Strategies for Improving the Outcome of Babies Born out of Hypertensive Disorders of Pregnancy’ was enlightening regarding newborns/preterm deliveries and the multiple interplaying factors. Numerous variables, including the mother's health state and the support of her family, as well as the interactions between these elements, affect the outcomes of babies born to women who have experienced preeclampsia. Increases in maternal blood pressure readings, early-onset preeclampsia and low birthweight poses higher risks [8]. Preeclampsia disrupts umbilical artery blood flow, causing placental insufficiency and often leading to iatrogenic preterm delivery [36]. Outcomes vary based on maternal condition, high blood pressure severity, prematurity, and intrauterine growth restriction. Preeclampsia reduces foetal survival with associated prematurity, low APGARs, NICU admissions, and mortality linked to increasing blood pressure [8]. Early-onset PE heightens risks of respiratory distress syndrome and asphyxia, while low birthweight infants may face cognitive challenges. Polycythaemia is also common in severe cases.

In Ghana, preterm birth rates are rising steadily. At the Korle- Bu Teaching Hospital (KBTH) preterm births rose from 9.3% in 2003 to 18.9% in 2019. Additionally, the Cape Coast Teaching Hospital observed an increase in the rate of preterm birth with overall survival at about 67.4% ( 91.6% for 34 weeks to 28% for < 28 weeks gestation) [37]. The key to better results is our comprehension of the present rates of newborn death. With every premature labour, clinicians must make a crucial decision on whether to deliver the patient early or transfer them to a perinatal care center while still in utero. This information is useful when advising families on the possible consequences of an early birth. Further, urgent strengthening of innovative strategies like adolescent care, antenatal care, effective health systems, and care services is needed. Continuous research and improved care are essential for better newborn outcomes.

#### Availability of HDP commodities in the public health sector

The findings from a desk review in 2020 examining availability of medicines for managing hypertensive disorders in pregnancy in Ghana’s public health sector showed multiple factors related to case management and prescriber behaviour in managing hypertension during pregnancy. Lower-level facilities managed a narrower range of HDP products compared to polyclinics and hospitals. Nifedipine tablet and methyldopa tablet were preferred for managing mild hypertension in pregnant women across all facility types. Notably, a significant number of facilities did not manage certain essential medications, including nifedipine, methyldopa, and magnesium sulfate injection, even though these drugs were within their level of care. Reasons for not stocking essential commodities varied, including the level of care, availability of alternative formulations, non-availability at supply points, and low incidence of HDP cases. Stockouts were reported in the three months preceding the survey, primarily due to non-availability at supply points, as confirmed by results from the national logistics management system. Many of the top brands of medications used for managing hypertensive disorders during pregnancy in Ghana are not registered with the Food and Drugs Authority-Ghana. This underscores the need for ongoing efforts to encourage pharmaceutical importers and manufacturers to register these essential products.

Challenges such as high inflation, delays in NHIS claims payments, and inadequate funding were highlighted as major barriers to ensuring consistent supply and procurement of essential HDP products. Prescriber preferences for managing severe hypertension also varied by facility type, with hydralazine injection being the most preferred in polyclinics and hospitals. However, challenges such as high inflation affecting the cost of HDP commodities, delays in NHIS claims payments, and inadequate funding at facility and regional levels were highlighted as major barriers to ensuring consistent supply and procurement of essential HDP products. It was recognized that Ghana’s Ministry of Health (MOH) should evaluate which categories of health facilities are permitted to handle calcium gluconate injection, an antidote for magnesium sulphate toxicity thereby ensuring facilities are equipped to promptly administer calcium gluconate injection, voiding missed opportunities to save lives.

Despite existing recommendations, modifications, and updates in the Standard Treatment Guidelines, the National Essential Medicines List, and the protocol for managing hypertensive disorders of pregnancy (HDP), many of these remain unimplemented. We advocate for the implementation of continuous development initiatives, such as compulsory annual online training courses, to raise awareness and promote the utilization of updated treatment protocols.

### Theme 3: leveraging on research to predict, recognize & manage high risk women

Exploration of this question considered the genetic risk factor of HDP, theAPOl1 Gene, the supplemental use of calcium to prevent preeclampsia, leveraging on research to predict, recognize & manage high risk women and sharing of power in priority- setting for health research projects. In addition, long term care and prevention of HDP were considered through a meaningful engagement session on ‘Prevention and Screening for Cardiometabolic Disease following HDP in Low-Resource Settings: A Systematic Review & Delphi Study’. Finally, to draw attention to the mental health of patients at risk of HDP and postpartum depression, the availability of psychosocial & mental health support was largely scrutinized through the oral abstract presentations which highlighted the need to incorporate the provision of such support in clinical practice.

#### Genetic risk factors of HDP; APol1 Gene

The gene of interest, APOL1, contains two common variants, G1 and G2, prevalent in sub-Saharan African and African American populations, providing protection against trypanosome parasites but increasing chronic kidney disease (CKD) risk. The distribution of these variants correlates with areas at risk for trypanosomiasis. Although G1 reduces severe disease risk, G2 can increase it, yet both increase CKD risk. APOL1's association with preeclampsia has been investigated, with future studies in Ghana aiming to replicate these findings. APOL1's association with preeclampsia prompted a study analyzing maternal and foetal genotypes. Results showed foetal genotype significantly increased preeclampsia risk in African American women. Collaboration by researchers from the University of Tennessee Health Science Center in Memphis, Tennessee and the University of Utrecht led to SPOT-BIO, studying HDP in Ghana. In Ghana, 25% carry APOL1 risk alleles with possible implications for a higher risk of preeclampsia. The study, conducted across referral hospitals, collects biospecimens and data, aiming to replicate findings and explore further genetic associations. This extensive study will offer insights into the complex relationship between APOL1, trypanosomiasis, and preeclampsia, potentially advancing understanding of genetic risks in maternal health.

#### The evidence to synthesis: the supplemental use of calcium to prevent preeclampsia

The lack of progress in reducing maternal mortality (MM) and morbidity from hypertensive disorders of pregnancy (HDP) necessitates urgent implementation of preventive measures. Calcium is the most extensively studied micronutrient for preventing preeclampsia [38, 39]. Calcium supplementation has shown promise in preventing preeclampsia (PE), supported by observational studies and randomized controlled trials [40]. Calcium has an anti-inflammatory effect and reduces endothelial activation and dysfunction in preeclampsia, possibly through the nitric oxide synthase pathway [39]. Evidence suggests that supplemental calcium intake can reduce the risk of preeclampsia in low-income countries [41]. WHO recommends 1.5–2.0 g of daily calcium for pregnant women at risk of PE [40]. However, global calcium intake remains inadequate, especially in Africa and Asia. Low calcium levels have been associated with PE severity in various studies, highlighting its importance in maternal health. The optimal calcium dosage for PE prevention is still debated, with medium doses showing effectiveness in low-risk populations and high doses in high-risk groups [39]. Calcium supplementation may reduce PE risk by inhibiting parathyroid hormone release and exerting anti-inflammatory effects. Despite evidence supporting calcium's role in PE prevention, challenges remain in scaling up supplementation programs to achieve significant population-level reductions in PE prevalence. Efforts to deliver calcium supplementation on a large scale, possibly through integrated antenatal care programs or fortification of staple foods, are crucial to achieve significant reductions in the population-level prevalence of preeclampsia.

#### Long term care: prevention and screening for cardiometabolic disease following HDP

Comprehensive screening and counselling are necessary to avoid chronic kidney diseases (CKD) and cardiometabolic disorders (CMD) after HDP [9]. Guidelines tailored for LMICs should be implemented, based on consensus. If CMD risk counselling is not started at the time of diagnosis, it should preferably start as soon as possible after delivery and continue either at the two-week review or before discharge. In order to help with risk identification and follow-up, obstetricians, midwives, and other maternity care providers should regularly advise HDP patients about the risks of CMD and CKD, using electronic medical records to facilitate risk identification and follow-up. Hospitals need to establish dedicated guidelines for HDP care continuity, including counselling for nontraditional CMD risk factors like gestational diabetes. Healthcare providers should be trained to understand the link between HDP and CMD/CKD. Basic instruments such as checklists can assist in ensuring comprehensive communication of risks to individuals who have experienced preeclampsia and are currently in the postpartum period. Lifestyle modifications such as healthy diet and regular exercise are recommended, with screening for CMD risk factors starting at six to eight weeks postpartum. Persistent proteinuria or hypertension at this stage warrants further assessment and possible nephrology referral. Subsequent screenings should occur at six months postpartum and annually thereafter, emphasizing maintenance of healthy BMI, lipid profiles, blood pressure, and fasting blood glucose levels. Both women and caregivers should be educated on these targets for optimal postpartum health.

#### Sharing of power in priority- setting for health research projects

Meaningful engagement in health research entails sharing decision-making power over setting health priorities and other phases of research projects [42]. It spans from active involvement of community partners in defining health priorities to ensuring their voices influence decisions. Tokenism, on the other hand, involves mere presence without diverse representation or influence in decision-making and this usually affects particularly disadvantaged and marginalised groups who often have the poorest health.

Strategies for meaningful engagement include recognizing social justice values, respecting diversity, and ensuring power sharing. To facilitate this, a toolkit has been developed by B Pratt [42] to guide research teams in sharing power with communities during priority-setting. This toolkit consists of four worksheets covering partner selection, decision-making, community engagement, and priority-setting design.

Research teams can use Worksheet 1 to consider and decide if they might benefit from having one or more additional academic or community partners in order to boost their efforts.

Worksheet 2 facilitates reflection and group decision-making on whether academic and community partners may share authority in their partnership. Worksheet 3 assists the study participants in considering and deciding as a group if broader community involvement is required for priority-setting and, if so, whether it can be accomplished in a meaningful way with members of a particular community. Research teams can then use Worksheet 4A to help them create a method for defining priorities for a particular global health research project.

Effective engagement requires addressing power imbalances, providing support to community partners, and framing partnerships with transparency and inclusivity. In research priority-setting, academic partners or research teams typically lead the translation of community consultations into research agendas. It is vital that they incorporate the viewpoints of community partners, especially those marginalized, to ensure their voices are heard and reflected in the research topics and questions. Ultimately, meaningful engagement empowers communities and ensures their voices are heard and valued in health research priority-setting processes. In situations where funding and constraints pose challenges, it's important to strategize how to present the priority-setting process and partnership in a way that attracts prospective partners and benefits their communities. Transparency about existing limitations prevents unrealistic expectations. Establishing a few fundamental components such as relational, environmental, personal, and normative elements is necessary before attaining genuine community participation. In order to facilitate community engagement, researchers and community partners can employ a variety of strategies, including matching systems, safe places, training, and meeting the requirements of a varied range of community partners.

#### The psychosocial & mental health support available to women at risk of HDP

In a highly religious, tribalistic and culturalist society like Ghana, pregnant women, mothers, and their families often go through traumatic pregnancies such as HDP with little to no psychosocial support due to various factors including cultural norms, religious myths, and stigmatization among many others [34]. Survivors also fail to patronize professional psychosocial support due to these same factors and this was demonstrated by the survivor stories shared in the patient documentary series Preeclampsia Diaries #1 [43]. As a result, most women and their families are harshly left to deal with their mental health during and after pregnancy with little structured support system to help them through the process of recovery from the trauma. Meanwhile the risk for postpartum depression (PPD) has also been found to increase in women with pregnancy and delivery complications including pregnancy-related hypertension [44].

The symposium deliberation and discourse affirmed this and revealed that the impact of HDP on perinatal mental health is understudied​. Globally the prevalence of Postpartum depression is estimated at 17% [45]. In Ghana the prevalence of postpartum depression (PPD) varies across studies, with estimates ranging from 8.4% to 24.3% [46]. Perinatal depression is a serious mental health disorder that can have negative consequences for mothers, infants, and families [47]. It was noted by Dr. Wuobar from a facility-based, descriptive cross-sectional research conducted from September to December 2021 that 9.39% of postpartum mothers with preeclampsia who were admitted or visited the child welfare clinics at the Eastern Regional Hospital in Koforidua, Ghana, had PPD. Hence, to espouse the need for a holistic psychosocial support system for pregnant women especially those who have pregnancy complications, Salim Wangabi and Amelie Nithimmar shared findings from their research on resilience and protective factors of maternal mental health in LMICs including Ghana (Tables 1 and 2).

**Table 1:** Risk factors

Sociodemographic factors	Clinical and pregnancy-related factors	Psychological and other risk factors
Poverty/ Low socioeconomic features	Unintended pregnancy	Intimate partner violence
Low education level	History of mental health problems	Insufficient emotional and social support by friends and (in-law) family
Multiparity	Complications during pregnancy	Insufficient emotional and social support by partner
Food insecurity	Poor physical health	Gender preference/ giving birth to a female
Being unmarried	History of abortion	
Being divorced	Perceived stress	
	Poor sleep quality

**Table 2:** Protective factors

Sociodemographic factors	Clinical and pregnancy-related factors	Psychological and other protective factors
High income	Planned pregnancy	High emotional and social support from friends and family
High education	Easy access to healthcare	Having a supportive partner
Null parity	Empathetic healthcare providers	Practical support with domestic tasks
Being married/ cohabited	Heightened postpartum care	Good relationship with mother
Being employed	Having knowledge about pregnancy	
	Good childcare facilities	
	Longer mandated rest postpartum	

These findings revealed that the social context of pregnant women cannot be overlooked because among the factors outlined such as the socioeconomic status, emotional, social, and practical support from family, partner, and friends were found to be particularly influential in adversely affecting or promoting maternal mental health. The role of the healthcare system and healthcare practitioners was also identified as very vital. Healthcare providers are encouraged to empathize with and be honest in their communication with pregnant women. The healthcare system should be overhauled to increase focus on the psychosocial aspect of pregnancy complications and develop corresponding interventions with a psychosocial approach including counseling before, during and after pregnancy.

At Institutional level Dr. Wuobar’s findings on the ‘*Prevalence and Determinants of Postpartum Depression among Women with Preeclampsia at the Eastern Regional hospital, Ghana*’ illustrated the need for partner support which is a significantly protective factor for Postpartum Depression (PPD); a lack of partner support carries a significantly higher risk of PPD.

The findings of this study showed that the prevalence of PPD within the subpopulation of women with preeclampsia is high and hence it should not be overlooked. However, the analysis indicated there is no single cause of PPD, but rather multifactorial determinants including stillbirths, marital status, educational level, and employment status. The need for routine screening for early identification of PPD among women with preeclampsia, provision of psychological support for mothers who have experienced pregnancy complications during and after delivery are recommended to improve the mental health of pregnant women and mothers. Healthcare providers are also encouraged to provide supportive care in addition to ensuring the standard of care for patients with preeclampsia.

## Key recommendations from the symposium

A multipronged strategy involving full expansion of the NHIS maternity coverage, a strengthened health system with greater state funding, sustained governmental commitment, surveillance efforts, reviews for preventability, updated versions of nationally agreed tools to support clinical decision-making and women centered care, and international cooperation are vital to addressing the clinical impacts of preeclampsia and improving outcomes in Ghana. Further strategies include:

1. Focusing future research endeavors on the main causes and physiological processes that underlie the clinical symptoms and results.
2. Training health professionals in perinatal mental health so they can promptly act to address poor perinatal mental health in women who experience pre-eclampsia
3. Prioritizing efficient resource management necessary for safe clinical care and eliminating bottlenecks in supply chain pathways.
4. Developing new patient provider partnership between maternity care providers and appropriate representative community bodies at regional/local level to enhance mutual understanding and cooperation, leading to better planning and oversight regarding preeclampsia care and support and pre-eclampsia research.
5. Implementing evidence-based practices to ensure timely and appropriate interventions for women with preeclampsia and improved clinical management.

## Conclusion

The symposium yielded intriguing insights regarding improving outcomes of hypertensive disorders of pregnancy as experts considered the drivers of and strategies to improving outcomes through shared decision-making, research and quality of care. The symposium underscored the value of teamwork among patients, researchers, and healthcare professionals. We can improve HDP management and results by engaging multi-sectoral and transdisciplinary perspectives, emphasizing collaborative decision-making and patient-centered communication, engaging in impactful research, and prioritizing high-quality maternal healthcare. The insights elaborated provide a roadmap for improved research, policy-making, and program decision-making for the future.

## Data Availability

Not applicable.

## Abbreviations

APEC: Action on Preeclampsia
EmONC: Basic Emergency Obstetric and Newborn Care
BP: Blood Pressure
CKD: Chronic Kidney Diseases
CMD: Cardiometabolic disorders
EPMM: Ending Preventable Maternal Mortality
GHS: Ghana Health Service
HDP: Hypertensive Disorders of Pregnancy
LMICs: Low- and Middle-Income countries
NHIS: National Health Insurance Scheme
PE/E: Preeclampsia/eclampsia
PPD: Post Partum Depression
PIERS: Preeclampsia Integrated Estimate of Risk
RMC: Respectful Maternity Care
SDG: Sustainable Development Goal
SPOT: Severe Preeclampsia Adverse Outcome Triage
UHC: Universal Health Coverage
WHO: World Health Organization
